# PReS-FINAL-2290: Is an abnormal lipid profile at diagnosis related to increased disease activity in JSLE patients

**DOI:** 10.1186/1546-0096-11-S2-P280

**Published:** 2013-12-05

**Authors:** C Thorbinson, L Watson, C Roberts, MW Beresford

**Affiliations:** 1 Institute of Child Health, University of Liverpool, Liverpool, UK

## Introduction

Lipid profile has been studied in Systemic Lupus Erythematous (SLE) due to the significant morbidity and mortality associated with premature coronary heart disease. Variations in lipid profile in adults with SLE are associated with overall disease activity, with Triglyceride (TG) levels associated with renal flare. Examination of lipid profile in juvenile-onset SLE (JSLE) showed a statistically significant drop in TG in transition from a state of active disease to inactive disease & TG levels to be significantly correlated with proteinuria at diagnosis.

## Objectives

To assess whether abnormal Total Cholesterol (TC), TG, High Density Lipoproteins (HDL) & Low Density Lipoproteins (LDL) levels are associated with increased disease activity at diagnosis in JSLE & whether TC & TG levels are associated with increased proteinuria and lupus nephritis (LN) at diagnosis.

## Methods

Analysis of prospectively collected data from patients diagnosed with JSLE taking part in the UK JSLE Cohort Study. Patients with a normal TC, TG, HDL & LDL levels at diagnosis were compared to those with abnormal results (defined by standard clinical laboratory ranges) using the global BILAG Disease Activity Score. TC and TG levels were compared in those with and without LN and proteinuria at diagnosis. Statistical analysis was done using the Mann-Whitney test. Continuous variables are presented as median (range) throughout.

## Results

79 patients were studied.There were no statistically significant differences in disease activity between patients with normal and abnormal lipid profile at diagnosis - see table 1 .

**Table 1:** 

Global BILAG Score (median ± IQR)	Patients with lipid concentrations in normal range	Patients with lipid concentrations in abnormal range	p value
Lipid Parameter			
TC	5 (0-37)	5 (0-40)	0.15
TG	2 (0-28)	5 (0-40)	0.30
HDL LDL	9 (0-37) 5 (0-40)	5 (0-40) 5 (0-28)	0.49 0.95

LN occurred more frequently in those with abnormal TG levels (n = 6; 17.1%) than those with normal levels (n = 1; 2.4%). Patients with LN at diagnosis have significantly increased TG levels (2.5(1.0-3.7)) compared to those without (1.3(0.5-8.1)), p = 0.001). However median TG levels in these groups were above the normal threshold. LN also occurred more frequently in those with abnormal TC at diagnosis (n = 9; 16.36%) as compared to normal TC (n = 0) and those with LN were found to have significantly increased TC levels (5.1(2.9-9.6)) compared to those without LN (4.2(1.7-7.9), p = 0.046). Median TC levels were above the normal threshold when LN was present and below the normal threshold when nephritis was absent.There was no statistically significant association between proteinuria and TC or TG at diagnosis.

## Conclusion

Abnormal lipid profile at diagnosis was not associated with significantly increased overall disease activity in keeping with previous paediatric but not adult lupus data. LN was more common in those with abnormal TC and TG levels at diagnosis in contrast to previous reports, and those with LN had significantly increased levels of TC and TG at diagnosis. There was no association between either TC or TG and proteinuria at diagnosis in contrast to previous studies. Further analysis of lipid profiles at diagnosis may help in the identification of LN in newly diagnosed SLE patients.

## Disclosure of interest

None declared.

